# Fatigue in Multiple Sclerosis: Assessing Pontine Involvement Using Proton MR Spectroscopic Imaging

**DOI:** 10.1371/journal.pone.0149622

**Published:** 2016-02-19

**Authors:** Wan Hazlin Zaini, Fabrizio Giuliani, Christian Beaulieu, Sanjay Kalra, Christopher Hanstock

**Affiliations:** 1 Department of Biomedical Engineering, University of Alberta, Edmonton, Alberta, Canada; 2 Department of Medicine, Division of Neurology, University of Alberta, Edmonton, Alberta, Canada; University of Pécs Medical School, HUNGARY

## Abstract

**Background/Objective:**

The underlying mechanism of fatigue in multiple sclerosis (MS) remains poorly understood. Our study investigates the involvement of the ascending reticular activating system (ARAS), originating in the pontine brainstem, in MS patients with symptoms of fatigue.

**Methods:**

Female relapsing-remitting MS patients (n = 17) and controls (n = 15) underwent a magnetic resonance spectroscopic imaging protocol at 1.5T. Fatigue was assessed in every subject using the Fatigue Severity Scale (FSS). Using an FSS cut-off of 36, patients were categorized into a low (n = 9, 22 ± 10) or high (n = 10, 52 ± 6) fatigue group. The brain metabolites N-acetylaspartate (NAA) and total creatine (tCr) were measured from sixteen 5x5x10 mm^3^ spectroscopic imaging voxels in the rostral pons.

**Results:**

MS patients with high fatigue had lower NAA/tCr concentration in the tegmental pons compared to control subjects. By using NAA and Cr values in the cerebellum for comparison, these NAA/tCr changes in the pons were driven by higher tCr concentration, and that these changes were focused in the WM regions.

**Discussion/Conclusion:**

Since there were no changes in NAA concentration, the increase in tCr may be suggestive of gliosis, or an imbalanced equilibrium of the creatine and phosphocreatine ratio in the pons of relapsing-remitting MS patients with fatigue.

## Introduction

Multiple sclerosis (MS) is an inflammatory disease of the central nervous system, which results in focal areas of demyelination. It is known to affect the white matter (WM) with axonal injury or loss as a common pathological feature occurring in the brain of MS patients [1]. This injury and resulting dysfunction has been observed to affect both normal appearing white matter (NAWM) and normal appearing grey matter (NAGM) even during the early course of the disease [2].

Fatigue is the most common and disabling symptom, experienced by 50 to 80% of MS patients [3]. Considering the significant impact of fatigue in MS patients, and despite the extensive research done to further our understanding of MS, the underlying pathophysiology of this symptom remains unknown [4].

In efforts to understand fatigue in MS, specific brain structures have been investigated using conventional magnetic resonance imaging (MRI), diffusion tensor imaging (DTI), and positron emission tomography (PET) to determine whether they are associated with this symptom [4,5]. One structure suggested to be involved with fatigue in MS is the pontine brainstem [6]. Originating within the pons is a complex network composed of several groups of projecting neurons, which makes up the ascending reticular activating system (ARAS). These groups of neurons diffusely project to many brain structures, including the neocortex, hippocampus, thalamus, hypothalamus and cerebellum [7]. The ARAS is responsible for arousal and sleep-wake cycle [8] and plays an important role in responding to stress [9], attention and behaviour [8]. These functions are often impaired in MS patients. It is thought that damage to structures of the ARAS in MS patients might impair its function in response to arousal and stimuli, thereby causing fatigue [6,10].

Magnetic resonance spectroscopy (MRS) serves as a complementary tool to MRI, and can be used to evaluate the neuronal integrity of the brain by examining the metabolite N-acetylaspartate (NAA). Such observations may infer the presence of disease in advance of anatomic changes seen when using conventional MR imaging methods. The signal from NAA is readily observed in proton MR spectra of the brain, and is found primarily in neurons. A decrease in the concentration of NAA in the pons, for MS patients with attention dysfunction, has been interpreted as resulting from neuronal damage, loss or dysfunction [11]. NAA signal measures have often been normalized to that from the total creatine signal (tCr–the sum of signals from creatine and phosphocreatine) to yield an NAA/tCr ratio. The concentration of tCr in MS brain tissue (excluding lesions) has been reported to be unaffected by the disease process when compared to controls [2]. However, the measurement of tCr is highly dependent on the WM:GM content being sampled due to a significant concentration gradient between these two tissue regions, due to the different neuron to glial density, where glial cells have a higher tCr concentration. It is also dependent on the experimental timing used for data acquisition. This latter dependence results from the fact that the tCr signal is derived from 2 metabolites which have different transverse relaxation times (T_2_ for Cr ~ 309 ms; and for PCr ~ 117 ms [12]). Therefore, as the TE for MR spectral acquisition increases, the measured t-Cr peak is more heavily weighted by the Cr signal, so any fluctuations in the Cr:PCr equilibrium may be observed as a change in the measured tCr peak relative to the other peaks. Such a fluctuation may be the result of a lower baseline level of PCr, or from an increase in activity in the brain region reducing PCr to maintain ATP levels. Clearly, these are important considerations when using the tCr signal as a reference metabolite when presenting MRS data as a ratio, for example NAA/tCr, even though this remains a common measure in the MRS literature.

While previous studies have used MRS to investigate changes in cerebral GM and WM [13], here we used multi-voxel proton MRS (chemical shift imaging–CSI^4^) to measure metabolite concentration changes for NAA and tCr in the pontine brainstem, which contains the ARAS nuclei. By carefully accounting for the differences in the tissue composition sampled (segmentation of each CSI voxel into GM:WM), and the partial volume dilution effects of the CSF on each of the measured MR spectra, we present a refined approach, which allows for estimation of intra-voxel as well as the standard inter-voxel metabolite concentration ratios. This was contrasted with the standard MRI determination of lesion load correlated with the clinical measure of Fatigue Severity Score (FSS), in order to add further evidence as to whether lesion load is a relevant factor in fatigue.

## Methods

### Participants

Nineteen women with relapsing remitting multiple sclerosis (RRMS) and low disability (ages 40 ± 7 years, range 27–56 years) and 18 healthy women (ages 40 ± 7 years, range 26–50 years) underwent fatigue screening and brain MRI/S (Table 1). Screening of the healthy controls ensured that none had suffered neurological or psychiatric conditions. This research study protocol was approved by the University of Alberta Health Research Ethics Board. Written informed consent was obtained from all participants.

**Table 1:** Characteristics of controls, low fatigue and high fatigue RRMS groups, and P-values from Mann-Whitney test between both patient groups.

Characteristics	Controls (n = 15)	Low Fatigue (n = 7)	High Fatigue (n = 10)	P-value
Mean Age (years)	38 ± 7 (26–49)	38 ± 5 (29–43)	42 ± 8 (29–56)	> 0.4
Median EDSS	-	1.5 (1.0–1.5)	1.8 (1.0–2.5)	> 0.2
Range Lesion Load (cm^3^)	-	0.15–16.25	0.44–37.17	> 0.8
Fatigue Severity Scale	18 ± 4 (13–26)	22 ± 9 (11–34)	52 ± 6 (42–59)	< 0.0001

MS patients were selected to have low-disability as measured by the clinical expanded disability status scale (EDSS) scores (median: 1.5, range: 0 to 2.5). Fatigue was assessed using the fatigue severity scale (FSS)[14]. The FSS is a self-administered test with nine statements that rates the severity of fatigue symptoms in the past week from a patient’s perspective; a value of 1 indicates strong disagreement and 7 indicates strong agreement with each statement. MS Patients were split into low (n = 9, FSS = 22 ± 10, range 11–34) or high (n = 10, FSS = 52 ± 6, range 42–59) fatigue (Table 1), where FSS scores more than 36 suggest that the patient is suffering from fatigue [14]. Fatigue and depression are highly associated in MS patients [4], but none of the patients included in this study had depression as evaluated using the Beck Depression Inventory (BDI I-II). Clinical assessments were done immediately prior to the MR scan and patient with clinical involvement of the cerebellum were excluded.

### Magnetic resonance image acquisition

**Fig 1:**
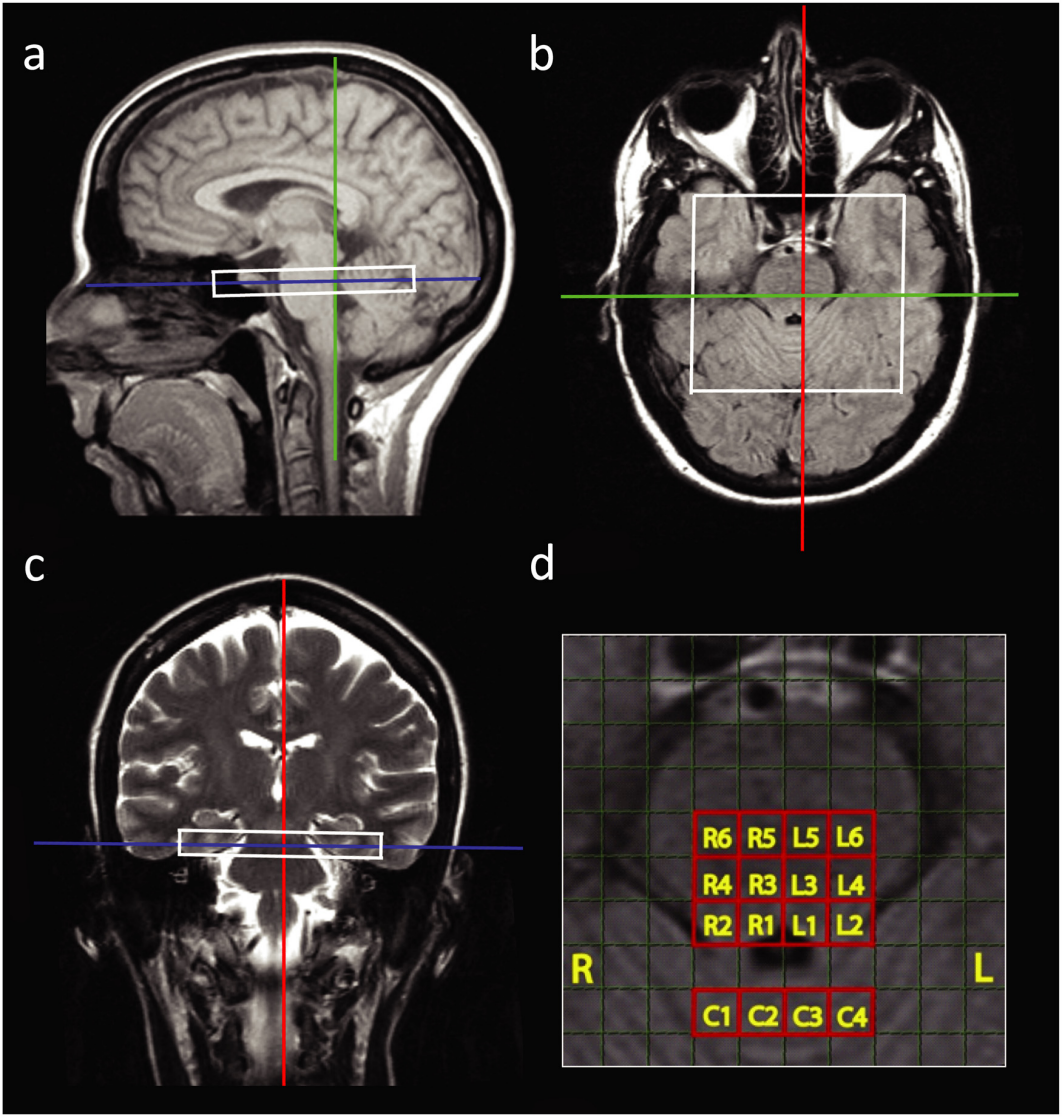
Field of view of the CSI volume (160x160x10 mm^3^) placement in one subject in sagittal (a), axial (b), and coronal (c) view. Zoomed in axial view of the pons (d) in one subject, sixteen CSI target voxels in the right (R1–R6) and left (L1–L6) and 4 reference voxels in the cerebellum (C1–C4). Each voxel size is 5x5x10 mm^3^.

MRI was acquired on a 1.5T Siemens Sonata scanner. Three sets of MR images were used to guide the CSI volume placement (Fig 1). First, a T_1_-weighted sagittal image was obtained to identify the brainstem, using the following parameters (slice thickness = 5 mm, TR = 199 ms, TE = 4.6 ms, flip angle = 90°, scan time = 0:50 min). Then an axial Fluid Attenuated Inversion Recovery (FLAIR) was placed perpendicular to the sagittal image and brainstem, to identify the fourth ventricle in the pons (slice thickness = 5 mm, TR = 9000 ms, TE = 106 ms, T1 = 2400 ms, scan time = 3:02 min). Finally, a coronal T_2_ image was obtained by placing it perpendicular to the previously described sagittal and axial images (slice thickness = 5 mm, TR = 7510, TE = 113 ms, scan time = 1:17 min).

### Chemical shift imaging

The ^1^H-MRS CSI data were acquired from a PRESS [15,16] localized region as a 16x16 matrix (zero-filled to 32x32). The water signal-suppressed CSI parameters were: slab thickness = 1 cm, FOV = 16 x 16 cm^2^, acquired volume = 8 x 8 cm^2^, CSI voxel dimension = 0.5 x 0.5 x 1 cm^3^, TR = 1750 ms, TE = 135 ms, 2 averages, flip angle = 90°, scan time = 15 min 10 sec.

### Structural images

A whole brain 3D T_1_-weighted MPRAGE was acquired for brain segmentation, with parameters as follows: TR = 1890 ms, TE = 4.89 ms, TI = 1100 ms, slice thickness = 1 mm, flip angle = 15°, FOV = 256x256 mm^2^, voxel dimension = 1x1x1 mm^3^, number of slices = 144, scan time = 4 min 38 sec. For segmentation analysis, the high-resolution T1-weighted image was aligned to the CSI volume through systematic visual inspection in MATLAB 7.8.0 (The MathWorks, Inc., Natick, MA). To quantify the composition of brain tissue in an individual CSI voxel, the T1-weighted image was segmented into gray matter (GM), white matter (WM) and cerebrospinal fluid (CSF) using SPM8 (Wellcome Department of Cognitive Neurology, London, UK). The percent GM in the brain tissue sampled, and the percent CSF in each CSI voxel, were calculated using the following eqs (1) and (2):

%GM =(100 × GM)⁄(GM + WM) (1)

%CSF =(100 × CSF)⁄(GM+WM+CSF) (2)

#### Determination of Lesion Load

Lesions were identified on the T2-weighted and whole-brain axial FLAIR images using an in-house semi-automated threshold intensity (MATLAB) to yield a total lesion volume per MS patient.

#### CSI data analysis

**Fig 2:**
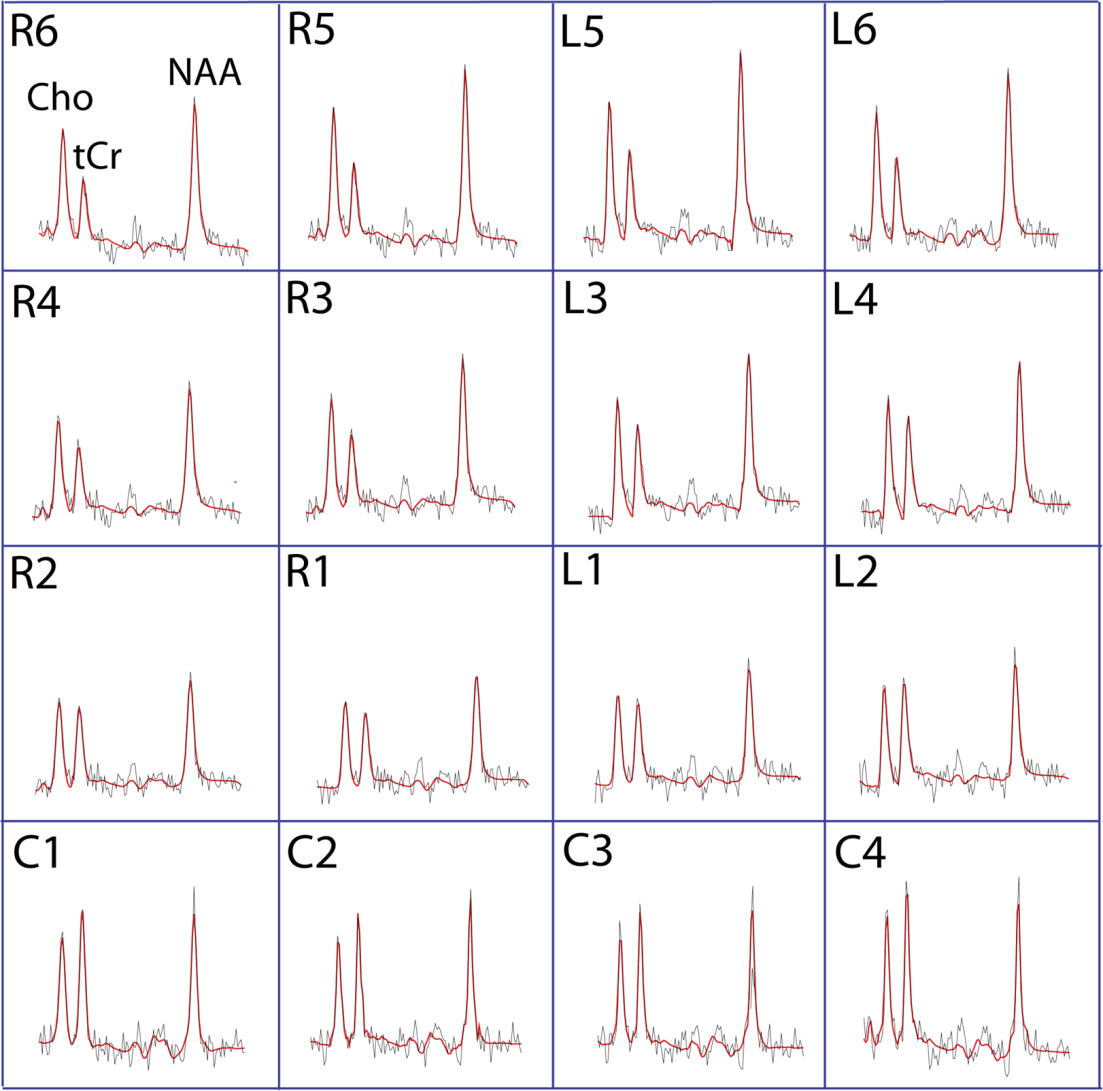
Spectra from 16 voxels in a 29 year old high fatigue patient. The red lines show the LCModel fit for each spectrum and the black lines are the raw spectral line.

The CSI data from the 32 subjects were analyzed using LCModel [17]. Sixteen voxels from the CSI matrix were selected for final analyses; six voxels were located in the right pons, six in the left pons and four in the cerebellum (Fig 1d). An example of a set of spectra from the sixteen voxels is illustrated in Fig 2.

Following LCModel analysis, spectral peaks with a Cramér-Rao lower bound (CRLB) of > 20% were excluded from further analyses [17]. Due to excessive patient motion 5 entire datasets were rejected (3 controls and 2 patients, leading to the final cohort of 17 MS patients and 15 controls). The tCr signal from a small number of voxels for the remaining 32 subjects failed to meet the CRLB limit and were rejected. The percentage of voxels, in which tCr data were rejected, amounted to 2.1% for controls, 4.5% for low fatigue (LF), and 4.4% for high fatigue (HF). Data included in the final analysis had similar mean CRLB for NAA in the pons {Control, LF, HF} = {7.4%, 7.8%, 8.0%} and cerebellum {Control, LF, HF} = {7.5%, 7.5%, 7.8%} for all three groups. Relative to NAA, the CRLB values were higher for tCr in the pons {Control, LF, HF} = {13.2%, 14.0%, 13.5%} and cerebellum {Control, LF, HF} = {9.0%, 9.5%, 9.8%}.

To correct for partial volume effect, we corrected each metabolite (NAA and tCr) concentration for CSF content in all 16 CSI voxels according to Eq (3) [18] below.

$$S\;=\left(100\;\times \;S_{\text{0}}\right)⁄\left(1-\left(\%CSF⁄100\right)\right)$$ (3)

where *S*_*0*_ is the uncorrected metabolite concentration in one voxel, CSF is the percentage of CSF content measured using Eq (2), and *S* is the corrected metabolite concentration in that voxel.

In addition to the NAA/tCr ratio calculated for the 16 voxels selected from the pons and cerebellum, the NAA and tCr values from voxels L1–L6, and R1–R6 were normalized to the average NAA or tCr that was measured for the 4 voxels in the cerebellum (Cb) (C1–C4). This yielded NAA/NAA_Cb_ or tCr/tCr_Cb_ ratios, which allowed us to examine whether changes in pons NAA or tCr were driving changes observed in the NAA/tCr ratio.

### Statistical analysis

Analysis of variance (ANOVA) with %GM as covariate was used to compare NAA/tCr ratio in the pons between all groups [19]. Only comparisons that reached statistical significance at p<0.05 after FDR correction are reported. NAA/tCr ratio comparisons that reached statistical significance underwent a subsequent group analysis of NAA/NAA_Cb_ and tCr/tCr_Cb_.

The Mann-Whitney test was used to compare lesion load, EDSS and age between high fatigue and low fatigue groups. Pearson’s correlation analysis was used to correlate MRS data with age, and fatigue scores (FSS) with disability measures (EDSS) and white matter lesion load (LL) in both patient groups.

## Results

**Fig 3:**
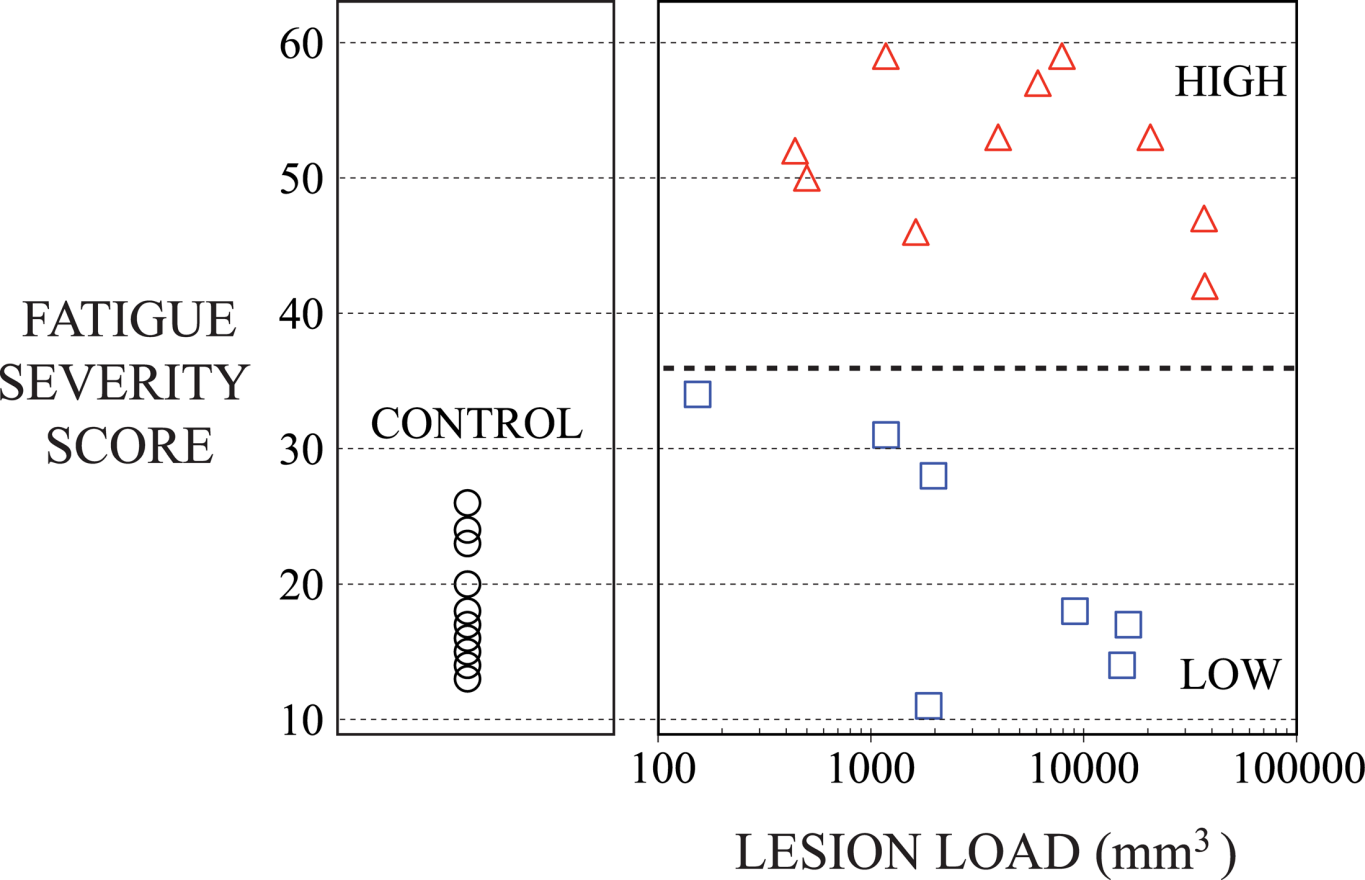
Plot of Fatigue Severity Score (FSS) versus Lesion Load in the right panel for MS subjects either with Low Fatigue (blue squares), or High Fatigue (red triangles). In the left panel the FSS for control subjects (black circles), is shown for comparison.

There was no significant difference between MS patients in low and high fatigue groups with respect to age and clinical EDSS (Table 1). Fatigue score was significantly different between MS patients in low and high fatigue groups (p < 0.0001) (Table 1); additionally fatigue score was not different between the low fatigue group and controls (22 ± 10 and 18 ± 5, respectively). Lesion load was highly variable between patients and was not significantly different between the low and high fatigue groups (Fig 3, Table 1). Correlation analysis over both RRMS groups combined did not reveal any significant association between EDSS, FSS and LL.

**Fig 4:**
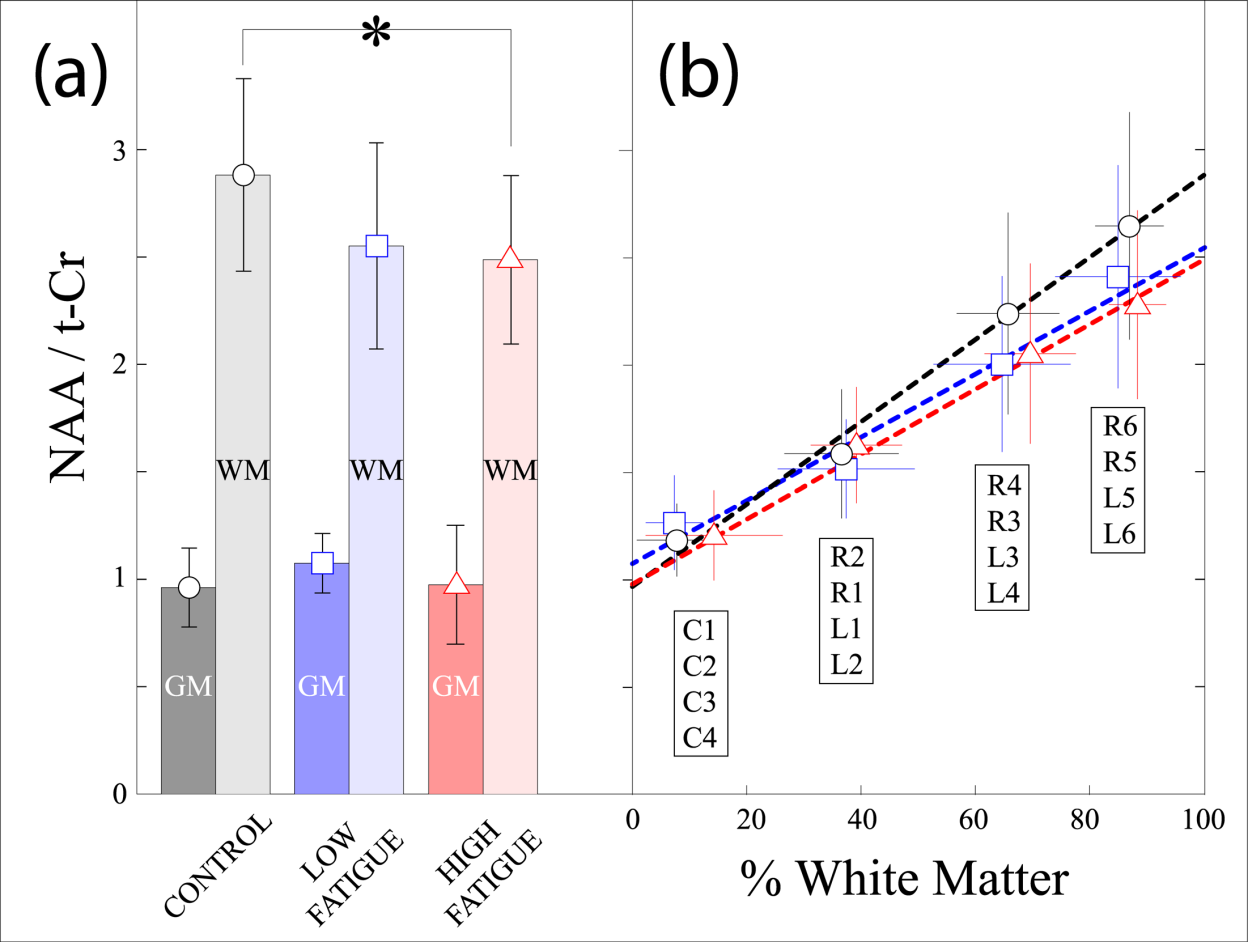
(a) Bar plots for control (black circle), low fatigue (blue square), and high fatigue (red triangle) subjects versus the NAA/tCr ratio, calculated for 100% gray matter (GM) and 100% white matter (WM). A statistically significant difference (p < 0.02) was found between the control and high fatigue WM data. (b) Plot of the mean and standard deviation values for %WM versus NAA/tCr for the 16 selected voxels in the CSI matrix located in the left/right pons (L1 –L6; R1 –R6) and cerebellum (C1 –C4), for control, low fatigue and high fatigue subjects.

Data for the NAA/tCr ratio and percent GM for all 16 voxels in each of the subject groups are shown in Table 2. In the pons, NAA/tCr was significantly smaller in L4, R5 and R6 in high fatigue when compared to controls and in L6 which was significantly smaller in low fatigue patients compared to controls. Further analysis of these data, presented in Fig 4, shows that when the NAA/t-Cr ratio is plotted against the %WM and projected to a composition of either 100% WM or GM, there is a significant difference in the NAA/t-Cr at 100% WM between the controls and HF group (Fig 4a, p < 0.02). The NAA/t-Cr curves for controls, LF and HF groups appear to converge as the %GM increases, illustrated in Fig 4b.

**Table 2:** Mean and standard deviation NAA/tCr and % Gray Matter (GM) data for the left/right pons (L1 –L6; R1 –R6) and cerebellum (C1 –C4) voxels selected from the CSI matrix for analysis. Significant changes are underlined for the NAA/tCr ratio between controls and marked with ‘☆’ for low fatigue or ‘★’ for high fatigue voxels (p < 0.05). No significant differences were observed for NAA/tCr, or for %GM, in voxels in the pons (white) or in the cerebellum (light gray).

Controls				Low Fatigue				High Fatigue			
**% GM**				**% GM**				**% GM**			
R6	R5	L5	L6	R6	R5	L5	L6	R6	R5	L5	L6
12.3 ± 5.7	14.3 ± 6.6	13.1 ± 6.8	12.4 ± 4.9	11.2 ± 10.4	16.2 ± 10.7	16.7 ± 10.3	16.0 ± 13.0	11.0 ± 5.4	11.3 ± 4.1	11.8 ± 4.6	12.3 ± 5.1
R4	R3	L3	L4	R4	R3	L3	L4	R4	R3	L3	L4
31.3 ± 7.8	34.9 ± 7.0	34.3 ± 9.1	36.5 ± 10.3	35.2 ± 9.4	34.8 ± 13.3	31.7 ± 12.0	39.5 ± 12.2	29.3 ± 8.4	32.5 ± 6.3	30.9 ± 9.7	28.8 ± 6.0
R2	R1	L1	L2	R2	R1	L1	L2	R2	R1	L1	L2
62.6 ± 8.9	63.6 ± 9.1	60.3 ± 8.7	67.2 ± 11.2	62.3 ± 14.5	62.6 ± 11.8	60.0 ± 10.1	63.5 ± 11.3	59.9 ± 8.1	60.1 ± 6.2	57.3 ± 8.8	65.8 ± 6.9
C1	C2	C3	C4	C1	C2	C3	C4	C1	C2	C3	C4
90.0 ± 8.3	94.3 ± 4.0	93.9 ± 5.2	90.7 ± 7.8	92.2 ± 4.4	94.7 ± 4.6	94.1 ± 4.6	89.6 ± 7.4	81.9 ± 14.5	89.8 ± 7.7	87.9 ± 8.8	83.3 ± 11.9
**NAA / tCr**				**NAA / tCr**				**NAA / tCr**			
**R6** ★	**R5** ★	L5	**L6** ☆	R6	R5	L5	**L6** ☆	**R6** ★	**R5** ★	L5	L6
**2.55 ± 0.50**	**2.84 ± 0.56**	2.69 ± 0.53	**2.52 ± 0.54**	2.53 ± 0.51	2.71 ± 0.32	2.50 ± 0.55	**1.91 ± 0.34**	**2.12 ± 0.29**	**2.32 ± 0.49**	2.36 ± 0.50	2.32 ± 0.47
R4	R3	L3	**L4** ★	R4	R3	L3	L4	R4	R3	L3	**L4** ★
2.09 ± 0.40	2.39 ± 0.51	2.38 ± 0.45	**2.11 ± 0.47**	2.01 ± 0.26	2.22 ± 0.32	2.07 ± 0.46	1.72 ± 0.44	1.82 ± 0.31	2.14 ± 0.50	2.35 ± 0.38	**1.91 ± 0.29**
R2	R1	L1	L2	R2	R1	L1	L2	R2	R1	L1	L2
1.48 ± 0.24	1.72 ± 0.33	1.72 ± 0.33	1.43 ± 0.23	1.40 ± 0.09	1.63 ± 0.26	1.66 ± 0.23	1.37 ± 0.20	1.44 ± 0.22	1.74 ± 0.26	1.80 ± 0.26	1.54 ± 0.19
C1	C2	C3	C4	C1	C2	C3	C4	C1	C2	C3	C4
1.20 ± 0.17	1.23 ± 0.17	1.20 ± 0.17	1.12 ± 0.16	1.29 ± 0.23	1.32 ± 0.26	1.29 ± 0.22	1.17 ± 0.19	1.19 ± 0.20	1.28 ± 0.27	1.24 ± 0.20	1.12 ± 0.17

**Fig 5:**
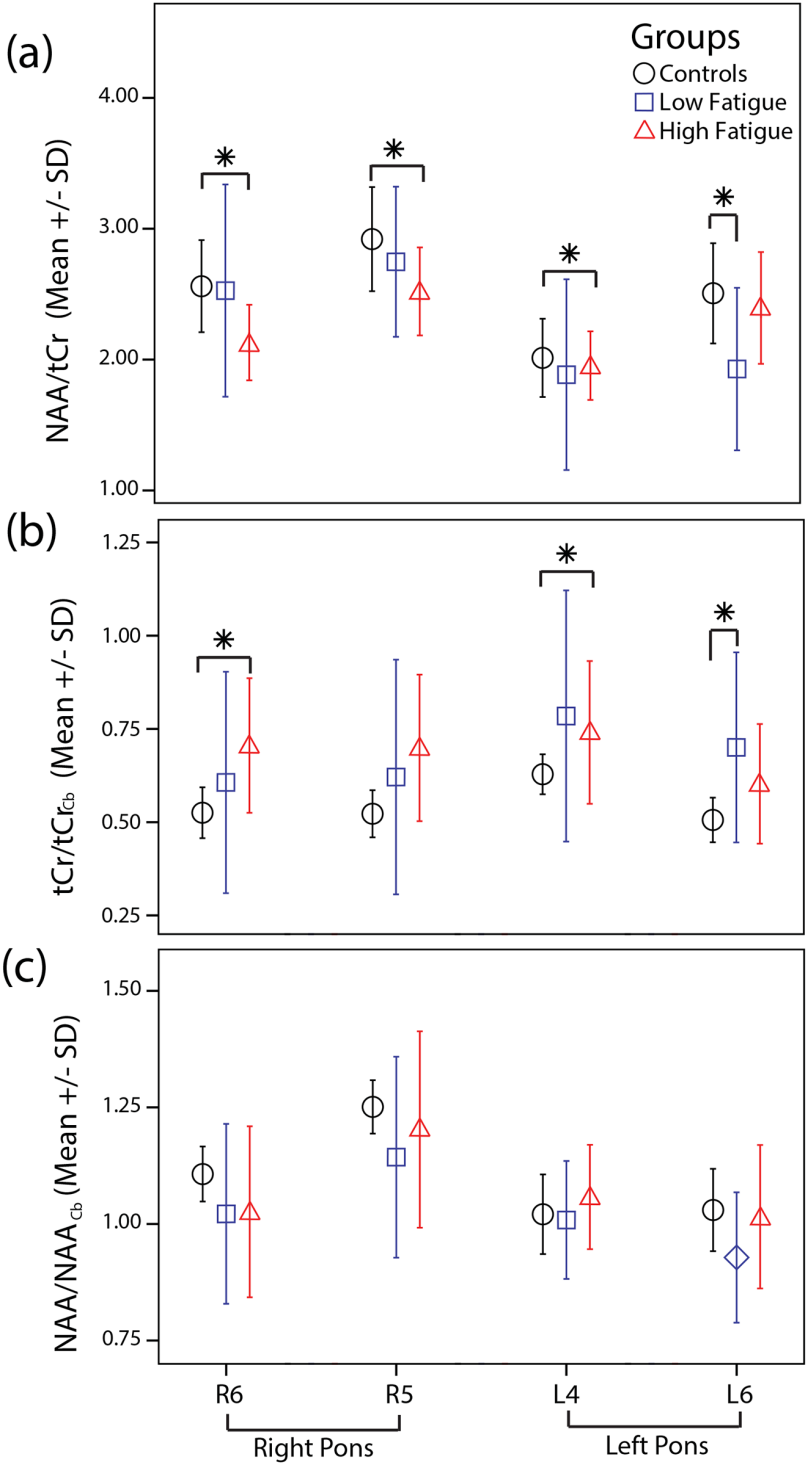
Mean plots showing the (a) NAA/tCr, (b) tCr/tCr_Cb_ and (c) NAA/NAA_Cb_ in voxels with significant difference group comparisons i.e. R6, R5, L4 and L6. Significant comparisons are marked with an asterisk (*).

The participants included in the study were only included if they had no cerebellar involvement. This was reflected in the NAA / tCr measures from the cerebellum which showed no significant differences between the study groups. However, the voxels located in the pons which showed a statistically significant difference in the NAA/tCr ratio, Fig 5a, were subsequently examined for individual metabolite changes relative to the cerebellum. This approach was used to provide an alternate reference source that was in the same CSI field, and yielded NAA/NAA_Cb_ and tCr/tCr_Cb_ ratios, shown in Fig 5b and 5c. The tCr/tCr_Cb_ ratio increased by 25% and 26% in L4 (0.75 ± 0.19, 0.60 ± 0.13, p = 0.039), and R6 (0.67 ± 0.20, 0.53 ± 0.10, p = 0.031), respectively, in HF compared to controls, but was not significantly different in R5 (0.64 ± 0.21, 0.53 ± 0.10, p = 0.11). Additionally, a 26% increase in tCr/tCr_Cb_ ratio was observed in L6 in LF compared to controls (0.63 ± 0.16, 0.50 ± 0.09, p = 0.042). There were no significant differences in the NAA/NAA_Cb_ ratio in any voxel when comparing subject groups. The analyses for tCr/tCr_Cb_ ratio were repeated by including subjects’ age as an additional covariate and yielded similar levels of significance.

## Discussion

Our CSI observations of lower NAA/tCr levels, which appears to be focused in the WM, illustrated in Fig 4, suggests an involvement of the tegmental pons in RRMS patients with higher levels of fatigue. Our further investigation using the NAA and tCr metabolites as references, indicated that the reductions in the NAA/tCr ratio were driven by increases in tCr, rather than reductions in NAA. The rationale for using the cerebellar NAA_Cb_ and tCr_Cb_ peaks as a reference is a means to establish which metabolite in the pontine NAA/tCr ratio was causing the observed decreases. While we screened the MS patients to ensure there was no cerebellar involvement, this does not directly imply that there were metabolic deficits in the volumes in the cerebellum we examined (C1:C4). However, the clear connection between the NAA/tCr ratio and the amount of WM (Fig 4), and that the cerebellar regions selected were composed predominantly of GM (~90%), we determined that the NAA_Cb_ and tCr_Cb_ peaks would indeed be appropriate as references, and allow us to calculate reliable NAA/NAA_Cb_ and tCr/tCr_Cb_ ratios for the pontine voxels. We acknowledge that caution is necessary, particularly for studies of MS brain, and that the percent of WM has to be considered for intra-voxel comparisons such as described here.

These observations may be caused by one of the following, either an increase in the glia:neuron cell ratio, or a disruption to the PCr:Cr equilibrium. The former, gliosis, occurs in MS lesions and would cause the tCr rich astrocytes and oligodendrocytes to occupy increased volume relative to the neurons in cerebral NAWM [19–24]. In tandem, the neuronal content would decrease, and should be accompanied by a decrease in the MRS marker NAA. Our observation that the NAA in the pons remained unchanged when referenced to cerebellar NAA possibly puts this hypothesis in doubt. However, glial proliferation in cerebral NAWM has been observed ex-vivo [20] which is in agreement with observations from histological studies [21, 25–26]. Absolute quantification of Cr and PCr using concurrent ^1^H and ^31^P spectroscopy in NAWM at the level of centrum semiovale (including parts of the corpus callosum) showed elevated tCr in MS patients possibly related to glial proliferation [27]. This study suggested an unchanged PCr:Cr ratio and unchanged NAA concentration in non-lesion voxels in patients compared to controls [27].

On the other hand, a disruption to the PCr:Cr equilibrium is consistent with recent studies on energy metabolism in MS. In the MR spectrum the PCr and Cr signals co-resonate at ~3.05ppm, and it has been shown that they can be estimated by using the difference in their transverse (T_2_) relaxation properties, where creatine’s T_2_ is significantly longer [12]. In the context of our observations, where our CSI spectral data are T_2_-weighted, shifting the equilibrium towards Cr would yield an increase in the apparent tCr signal. Such a disruption is supported by a ^31^P spectroscopy study, where increased PCr in MS patients was suggested to represent metabolic dysfunction in the NAWM [22,23]. An alternate metabolite, myo-inositol (mI), would be useful to resolve whether the changes in tCr result from gliosis or from a disruption in the PCr:Cr equilibrium. The utility arises from the knowledge that mI is found predominantly in glial cells. However, due to the CSI timing conditions used in our study, the mI signal was too low to allow quantification because of the J-coupling evolution effects on signal yield. This would be an important amendment to a future study protocol.

Many theories and hypotheses have been formulated to understand the underlying pathophysiology of fatigue. Damage to the ascending reticular activating system (ARAS) has been proposed to play a role in fatigue [10,24]. The ARAS originates in the brainstem and is connected to the neocortex (projecting frontally), thalamus and hypothalamus [28]. It was suggested that dysfunction from within the originating region of the ARAS, in particular the tegmentum of the upper pons and midbrain, could influence functions of the down stream sites [6]. In our study, tCr increase was observed in the upper tegmental pons in high fatigue MS patients. A previous spectroscopy study suggested axonal damage in the right rostral dorsal pons in MS patients with selective attention deficit and low disability [29]; fatigue and attention is also known to be highly associated in MS patients [4], but attention was not measured in our cohort. In addition, our study shows for the first time an involvement of the tegmental pons in area ventral and lateral to the location of the locus coeruleus. This area contains the pedunculopontine nucleus. This nucleus has been shown to be associated with significant arousal mechanisms. Hypercativation of the cells within this nucleus can cause insomnia [30]. On the other hand, neurons from this nucleus seem to be be the site of action for a drug used to treat narcolepsy, modafinil [30]. Modafinil is also used to treat fatigue in MS patients [31] and it has been recently shown to be effective in alleviating post-stroke fatigue [32].

Although there have been some preliminary reports about the correlation of fatigue with gray matter atrophy and lesion load on MRI [33], it is still not clear whether the location of the lesions or the regional atrophy play a role. It has been shown that MS fatigue is associated with the diffuse disruption of pathways within the parietal and frontal lobes [34]. The same pathways could be disrupted by injury affecting brainstem nuclei with diffuse projections to the cortex and gray matter nuclei as the one we describe in the current study. It has also been suggested that the underlying cause of fatigue is the functional and structural disconnection between the frontal cortex and the deep gray structures including basal ganglia and thalamus [35]. An MRS study attributed dysfunction of the basal ganglia, specifically of the lentiform nucleus, to the development of fatigue in MS patients [36]. A significant correlation was found between altered white matter integrity, as assessed by diffusion imaging, of the frontal cortex with its projections to the occipital, striatal, frontal and limbic network with fatigue perception in their MS patients [35]. A functional MRI study observed increased activations in the frontal lobe, thalamus and caudate [37]. Together, all these observations suggest that the underlying mechanism of fatigue in MS is not focal, but rather involves many diffuse inter-connected brain regions. The lack of significant correlation between fatigue and lesion load adds further evidence to diffuse involvement, however, lesion location remains important. For example, injury to nuclei which project diffusely would likely give rise to a diffuse effect albeit from a focal lesion.

## Conclusion

In conclusion, proton MRS was used to study changes in the relative concentrations of metabolites in the upper pontine brainstem in relapsing-remitting MS patients with low disability that experience a range of levels of fatigue. A lower NAA/tCr ratio was measured in several regions of the pons, and the change in this ratio was driven by elevation of the tCr levels in high fatigue patients compared to controls. Moreover, this tCr change was focussed in the WM regions in particular in an area of the pontine tegmentum containing the pedunculopontine nucleus. Increased tCr levels possibly reflect increased glial cell numbers in those NAWM regions or may suggest an energy metabolic dysfunction in patients with fatigue. The study also points to two methodological issues, which should be considered when performing MR spectroscopy. First, it adds to the increasing evidence that caution is warranted in the use of tCr as an internal concentration reference, and this is particularly relevant in the study of MS. Second, that consideration of the proportion of white matter and gray matter in the selected voxels has a profound effect on the metabolite measurements, and when comparing a single metabolite from different voxel locations the CSF contribution needs to be accounted for. Finally, the estimation of lesion load using MRI, and its lack of correlation with fatigue, adds further evidence to MS being the result of a diffuse rather than focal effect.
